# No Evidence for a Relationship Between Hair Testosterone Concentrations and 2D:4D Ratio or Risk Taking

**DOI:** 10.3389/fnbeh.2018.00030

**Published:** 2018-03-05

**Authors:** Richard Ronay, Leander van der Meij, Janneke K. Oostrom, Thomas V. Pollet

**Affiliations:** ^1^Department of Leadership and Management, Faculty of Economics and Business, University of Amsterdam, Amsterdam, Netherlands; ^2^Department of Industrial Engineering, Eindhoven University of Technology, Eindhoven, Netherlands; ^3^Department of Management & Organization, School of Business and Economics, Vrije Universiteit Amsterdam, Amsterdam, Netherlands; ^4^Department of Psychology, Faculty of Health and Life Sciences, Northumbria University, Newcastle, United Kingdom

**Keywords:** testosterone, cortisol, hair samples, 2D:4D ratio, risk taking, dual hormone hypothesis

## Abstract

Using a recently developed alternative assay procedure to measure hormone levels from hair samples, we examined the relationships between testosterone, cortisol, 2D:4D ratio, overconfidence and risk taking. A total of 162 (53 male) participants provided a 3 cm sample of hair, a scanned image of their right and left hands from which we determined 2D:4D ratios, and completed measures of overconfidence and behavioral risk taking. While our sample size for males was less than ideal, our results revealed no evidence for a relationship between hair testosterone concentrations, 2D:4D ratios and risk taking. No relationships with overconfidence emerged. Partially consistent with the Dual Hormone Hypothesis, we did find evidence for the interacting effect of testosterone and cortisol on risk taking but only in men. Hair testosterone concentrations were positively related to risk taking when levels of hair cortisol concentrations were low, in men. Our results lend support to the suggestion that endogenous testosterone and 2D:4D ratio are unrelated and might then exert diverging activating vs. organizing effects on behavior. Comparing our results to those reported in the existing literature we speculate that behavioral correlates of testosterone such as direct effects on risk taking may be more sensitive to state-based fluctuations than baseline levels of testosterone.

## Introduction

Although studies have documented a positive relationship between testosterone and risky economic decisions, the evidence has been inconsistent, with linear (Apicella et al., 2008), non-linear (Stanton et al., 2011) and null relationships (Zethraeus et al., 2009). One explanation for these inconsistencies could be the failure to distinguish between measurements of state-based levels of testosterone and the measurement of more trait-like (baseline) levels of testosterone. The majority of studies exploring the relationships between testosterone and risk taking have measured state-based levels of testosterone via saliva samples. This lends itself to experimental studies seeking to test the contextual role of fluctuations in testosterone on behavior. However, studies that aim to test for relationships between baseline endogenous testosterone levels are potentially confounded by these same contextually bound fluctuations when using saliva samples. In the current study we measure testosterone using a recently developed alternative assay procedure in which hormone levels are assayed from hair samples. Hair samples should provide a stronger test of the relationship between baseline levels of testosterone and risk taking, as hair samples indicate average fluctuating testosterone levels across 3 months and thus filter out contextual noise in hormone measurements. As per the Dual Hormone Hypothesis (Mehta and Josephs, 2010), we test both the direct effect of hair testosterone concentrations on risk taking and its interaction effect with hair cortisol concentrations. Contributing to the research on the relationships between different hormone measurements, we also examine the relationship between hair sample testosterone and an often used measure of prenatal testosterone, the 2D:4D ratio—the relative length of the index finger (2D) and the ring finger (4D) (Manning, 2002).

Two influential and complementary theoretical models that have been offered as explanatory frameworks for understanding the dynamic relationship between testosterone and social behavior are the Challenge Hypothesis (Wingfield et al., 1990; Archer, 2006) and the Biosocial Model of Status (Mazur, 1985; Mazur and Booth, 1998). The Challenge Hypothesis posits that testosterone motivates resource and mate-seeking behaviors, including those associated with aggression and competition, when the social context deems such behaviors as *reproductively beneficial* for the organism. Similarly, the Biosocial Model of Status states that testosterone encourages competitive behaviors that serve the function of increasing status. In support of these frameworks, testosterone has been repeatedly linked to competitive, dominance- and status-seeking behaviors in human and non-human males. For instance, the males of many species show increased competitive behaviors during breeding season when testosterone levels are known to peak (Harding, 1981; Balthazart, 1983; Wingfield et al., 1990; Denson et al., 2013), with similar hormonal (Van der Meij et al., 2010) and behavioral (Ronay and von Hippel, 2010) responses to mating competition among human males (for a review in humans, see Eisenegger et al., 2011).

One way in which testosterone might fuel competition is via an increased tolerance for risk. Although the literature does not offer a consistent picture of the relationship between endogenous testosterone and risk taking, a number of studies have reported positive relationships. For instance, Apicella et al. (2008) reported a positive linear relationship between testosterone and financial risk taking in a sample of Harvard undergraduate men. Similarly, Coates and Herbert (2008) reported a positive relationship between testosterone and the day to day returns of London financial traders. Sapienza et al. (2009) found a positive relationship between testosterone and risk taking for women, though not men. Ronay and von Hippel (2010) reported that adult male skateboarders’ testosterone levels, measured in the context of sexual competition primed by the presence of an attractive female experimenter, are positively associated with physical risk taking. Last, Stanton et al. (2011) found a non-linear relationship—both low and high testosterone predicted greater risk taking—among men and women. Taken together, the empirical evidence suggests an intriguing but inconsistent relationship between testosterone and risk taking.

Similarly, the published work exploring the relationship between exogenously administered testosterone and risk taking consists of a small collection of intriguing but inconsistent findings. Although two administration studies involving only women found no evidence for a causal relationship between testosterone and economic risk preferences (Zethraeus et al., 2009; Boksem et al., 2013), testosterone administration has been shown to increase women’s risk taking on the Iowa gambling task (Van Honk et al., 2003). However, another study involving pharmacological manipulations in men found that higher testosterone levels were associated with increased risk seeking as measured via the balloon analog risk task (BART; Lejuez et al., 2002), but not in the Iowa gambling task or a dice task (Goudriaan et al., 2010).

Although results are mixed, the theoretical foundations (Mazur, 1985; Wingfield et al., 1990; Mazur and Booth, 1998; Archer, 2006) that have inspired these empirical tests seem sound, and comparative studies among non-human animals (Rose et al., 1971; Rada et al., 1976; Harding, 1981; Schwabl and Kriner, 1991; Wingfield and Hahn, 1994) provide corroborating support for a relationship between testosterone and competitive behaviors in general. Ancillary evidence is also suggestive of such a positive relationship. For instance, men’s higher testosterone levels relative to women (e.g., Pollet et al., 2011; Ronay and Carney, 2013), and a robust age-related decline in testosterone (Harman et al., 2001) map onto reliable sex differences in risk taking (Byrnes et al., 1999; Ronay and Kim, 2006), and age-related declines in risk taking (Kaufman and Vermeulen, 2005). The inconsistency of the empirical work therefore represents something of a puzzle for researchers seeking to understand the behavioral effects of testosterone.

Testosterone not only has activating effects that emerge from endogenous circulating levels of the hormone, but prenatal testosterone also manifests organizing effects that shape how the brain and body develop (Manning, 2002). One putative marker of *in utero* androgen exposure is the 2D:4D ratio, with lower ratios indicating exposure to higher levels of androgens during prenatal development (Manning, 2002). Lutchmaya et al. (2004) examined the relationship between the 2D:4D ratios of 33 children at age two, and the level of fetal testosterone (measured via amniocentesis) they were exposed to during the second trimester of their gestation. They reported a strong negative relationship between digit ratios and fetal testosterone levels.

Evidence for a negative relationship between 2D:4D ratio and endogenous levels of circulating testosterone during adulthood is less persuasive. Although Manning et al. (1998) report a significant negative relationship between 2D:4D ratio and endogenous testosterone levels of 58 men, further investigations (Campbell et al., 2010; Sanchez-Pages and Turiegano, 2010) have been unable to reproduce this effect and a meta-analysis (Hönekopp et al., 2007) also suggests no robust effect. Nonetheless, the conceptual overlap between the two measures has motivated a number of researchers to examine the behavioral effects of 2D:4D ratio in contexts where theory suggests testosterone should play a role, with conceptually consistent results (Bailey and Hurd, 2005; Van den Bergh and Dewitte, 2006; Voracek et al., 2006; Millet and Dewitte, 2009; Ronay and von Hippel, 2010; Ronay and Galinsky, 2011; Ronay et al., 2012). Irrespective of the likely surfeit of failed studies in this vein that remain buried in file drawers, the conceptual consistency between the effects of 2D:4D ratio and testosterone on behavior, coupled with the lack of empirical support for a reliable relationship between the two produces yet another puzzle of interest. To explore one possible solution to this puzzle, we turned our attention to the method by which testosterone levels are most commonly measured.

Testosterone levels vary across the day (Granger et al., 1999) as well as in response to a range of social contextual factors (Mehta and Josephs, 2006; Van der Meij et al., 2008, 2010). Endogenous testosterone levels vary even in response to partisan alignment following presidential election outcomes (Stanton et al., 2009), and football team affiliation following match day (Van der Meij et al., 2012). This has obvious advantages for researchers seeking to test the contextual role of fluctuations in testosterone on behavior (e.g., Ronay and von Hippel, 2010; Apicella et al., 2014), such as would be predicted by The Challenge Hypothesis (Wingfield et al., 1990; Archer, 2006) and the Biosocial Model of Status (Mazur, 1985; Mazur and Booth, 1998). However, studies seeking to test the relationships between *baseline* endogenous testosterone levels and other variables—such as 2D:4D ratio and risk taking—are disadvantaged by these same contextually bound fluctuations. This problem is exacerbated by the fact that much of the published research, samples testosterone levels at a single time point, rather than via multiple measures that might lead to a more accurate and stable measure of baseline testosterone. Thus, one possible contributing factor to the inconsistent effects of testosterone on risk taking, and the relationship between 2D:4D ratio and circulating testosterone, may be the failure to distinguish between measurements of state-based levels of testosterone—such as are derived from single time point measures—and the more stable, trait-like levels of testosterone—such as might be captured by aggregating across multiple time points.

Mehta and Josephs (2010) have proposed the Dual Hormone Hypothesis, which posits that testosterone’s role in status-relevant behavior should depend on concentrations of cortisol, a hormone that is released in response to physical and/or psychological stress. Specifically, the Dual Hormone Hypothesis predicts that behavioral effects follow from an interaction between testosterone and cortisol—testosterone should be positively related to status-seeking behaviors only when cortisol concentrations are low. According to the model, when cortisol concentrations are high, status-seeking behaviors should be inhibited. The predictions of the model have been demonstrated on a range of dependent variables including risk taking (Mehta et al., 2015), self-reported aggression (Popma et al., 2007; Denson et al., 2013) and retrospectively in juvenile crime (Dabbs et al., 1991). However, in keeping with the majority of the endocrinological literature, these tests of the Dual Hormone Hypothesis have relied upon isolated single time point measures of both testosterone and cortisol.

The goal of the current research was to reexamine the relationships between baseline testosterone, 2D:4D ratios, and risk taking, using a recently developed alternative assay procedure in which testosterone levels are assayed from hair samples using an liquid chromatography tandem mass spectrometry method (LC-MS/MS)-based method. We measured cortisol simultaneously so as to test for possible interacting effects of testosterone and cortisol on risk taking, as per the Dual Hormone Hypothesis (Mehta and Josephs, 2010). As testosterone (Johnson et al., 2006; Ronay et al., 2017) has been suggested to facilitate higher levels of overconfidence, and overconfidence has been linked to risk taking (Miller and Byrnes, 1997; Camerer and Lovallo, 1999; Campbell et al., 2004; Malmendier and Tate, 2008) we also measured participants’ overconfidence in order to examine the possibility of these relationships with hair testosterone concentrations.

## Materials and Methods

### Participants

Participants were 162 non-psychology students (53 male, 109 female; *M*_age_ = 22.05, *SD*_age_ = 2.85) from the Vrije Universiteit Amsterdam. Participants received 8 € for their participation. Prior to analysis we made a decision to exclude 14 participants due to incomplete measures or measurement error. Initial analysis of the hair samples revealed five cases to be outside of known measurement limits, suggesting unacceptable noise in the assaying, and so these cases were excluded from further analyses. Three further cases reported medical histories known to directly affect hormones (Polycystic ovary syndrome, Betamethason medication and cancer treatment), and so these too were excluded from further analyses (Granger et al., 2009). This yielded a final sample of 140 participants (43 male, 97 female; *M*_age_ = 21.93, *SD*_age_ = 2.88). We acknowledge that our final sample size for males is less than our initial goal of 100 males and 100 females, thus tempering the strength of our conclusions.

### Procedure

The study was approved by the Scientific and Ethical Review Board (VCWE) of the Vrije Universiteit Amsterdam. Participants first read an informed consent form and provided written consent for their participation. Participants then provided demographic and health information. To assess risk taking, participants completed the BART (Lejuez et al., 2002). In addition, they completed measures on self-esteem, personality, and sexual behavior, which are not the focus of the current research and thus not discussed here. Participants were then asked to position their hands palm down on a flatbed scanner so as to allow us to capture images of both hands for determining 2D:4D ratios. Finally, hair samples were taken and participants were debriefed and paid.

### Measures

#### Hair Samples

Testosterone and cortisol concentrations were determined from hair samples with a LC-MS/MS. This method is considered to be a reliable and precise way to measure testosterone and cortisol concentrations (Gao et al., 2013). Specifically, for these hormones, intra- and inter-assay coefficients of variation are between 3.1% and 8.8% and the limits of quantification (LOQ) are below 0.1 pg/mg (Gao et al., 2013). Hair sampling was done according to the instructions of the laboratory of Biological Psychology at the Technical University of Dresden. Three hair strands were cut with scissors as close as possible from the scalp from a posterior vertex position and tied with a thread. Hair strands were placed in aluminum foils that were put in envelopes. The envelopes were placed in a specially prepared box and sent to the laboratory of biological psychology at the Technical University of Dresden (Germany) for analyses. Steroid concentrations were determined from hair segments 3 cm closest to the scalp, which represents hair grown over the last 3 months prior to sampling when assuming an average hair growth of 1 cm per month (Wennig, 2000).

#### 2D:4D Ratio

The lengths of the second and fourth digits were independently measured by two master’s students, from the ventral proximal crease of the digit to the tip of the finger using the “Measure” tool in Adobe Photoshop. Digit ratios were calculated by dividing the length of the 4th digit on the hand by the length of the 2nd digit on the same hand (Manning et al., 1998). Measurements were computed in the absence of any other information about the participant. The correlation between the measurers was >0.99.

#### Risk Taking

Risk taking was assessed via the BART (Lejuez et al., 2002). The BART has been shown to possess good test-retest reliability (White et al., 2008) and has been validated against self-reported correlates of risk taking, including psychopathy (Hunt et al., 2005), impulsivity and sensation seeking (Lejuez et al., 2002). Critically, the BART has also been shown to predict a number of real-world risk taking behaviors including cigarette smoking, alcohol use, illicit drug use, gambling and sexual risk taking (Lejuez et al., 2002, 2003; Hopko et al., 2006).

The BART is a computer task in which participants are presented with a series of 30 onscreen balloons and a virtual “pump” that when clicked incrementally expands the size of the current balloon until a randomly determined pop point is reached and the balloon explodes. Participants were presented with a series of 30 balloons and not just a single balloon to increase the reliability of our measurement. Participants were instructed that with each additional pump they would earn 1 cent that would accumulate in a temporary bank, also on screen. However, when a balloon was inflated past its pop point, the balloon exploded and all money earned on that particular balloon would be lost. To guard against this risk, participants could choose to stop at any point by clicking on a “Collect $$$” button, also onscreen, at which point the money in the temporary bank would be transferred to a permanent bank. The probability that a balloon would explode increased incrementally with each pump—1/128 for the first pump, 1/127 for the second pump, etc., the probability of an explosion on the 128th pump was therefore 1/1. According to this algorithm, the average breakpoint was 64 pumps (Lejuez et al., 2002). Participants received onscreen instruction before the test started but did not receive any information about the probability of the explosion, neither at the start or during the task. Thus, the game creates a tension between securing one’s accumulated winnings, against the pursuit of further, albeit diminishing relative returns. As our goal was to measure risk taking behavior and not hypothetical or self-reported risk attitudes, which might capture diverging aspects of risk taking (Battalio et al., 1990; Holt and Laury, 2005; Harrison, 2006; Branas-Garza et al., in press), participants were informed that they would be paid 10 percent of their winnings at the conclusion of the experiment (*M*_euro_ = 0.76, *SD* = 0.21). However, as this is a rather minimal stake, which may incentivise riskier decisions than in real life (Holt and Laury, 2005), we decided to also inform participants that the participant who accumulated the most money on the BART (30 balloons, across all sessions) would receive a cash prize of 50 € once testing was concluded. Together, these incentives were intended to parallel real world risk taking decisions in which risk taking is rewarded up until a point, after which further riskiness results in poorer outcomes. All participants were paid accordingly. Each participant was presented with 30 virtual balloons and as recommended (Lejuez et al., 2002) the average number of pumps on all unexploded balloons served as our dependent variable.

#### Overconfidence

Overconfidence was operationalized as overestimation of one’s actual performance (Fischhoff et al., 1977; Kruger and Dunning, 1999; Kruger and Mueller, 2002; Larrick et al., 2007; Moore and Healy, 2008) on an existing General Knowledge Questionnaire (GKQ; Michailova, 2010). We used a previously adapted version (Ronay et al., 2017) of the GKQ (Michailova, 2010; Michailova and Katter, 2014), taking the 18 items from Michailova’s (2010) original measure (e.g., *How many days does a hen need to incubate an egg?*) and adding six further items (Ronay et al., 2017). Participants were instructed to choose the correct answer from three alternatives and to provide a number between 33% (chance) and 100% (absolute certainty) indicating their confidence in the accuracy of that answer. Consistent with previous work and as many scholars recommend^1^, we computed overconfidence by regressing participants’ confidence scores (i.e., mean confidence ratings) onto their accuracy (i.e., percentage of correctly answered items) and saving the standardized residual scores (DuBois, 1957; Cronbach and Furby, 1970; John and Robins, 1994; Cohen et al., 2003; Anderson et al., 2012). This approach isolates the variance in participants’ confidence while controlling for variance in accuracy—i.e., confidence over and above accuracy.

### Statistical Analyses

Our analysis plan was registered on osf.io: 4h3cd. We analyzed male and female data separately as the distribution markedly differs between the sexes (Stanton, 2011). Given the skewness we performed a log transformation for testosterone and cortisol concentrations for our core analyses. The analysis plan fully details the analytical strategy as well as the robustness checks employed. Our key analyses are Bayesian Regression Models via the “BRMS” package in R (Buerkner, 2015). The estimation was based on four chains, each containing 2000 iterations (1000 for burn-in) using non-informative priors on all model parameters. We examined convergence via Rhat (close to 1; see ESM) and evaluated model fits via information criteria (WAIC, LOOIC) compared to a null model (intercept only; Vehtari et al., 2017). These differences between models in terms of fit can be roughly interpreted according to the following rules of thumb: with a difference (Δ) of 1–2 units offering little to no support over a null, between 4–7 units offering considerable support for an alternative model, and those with >10 units offer full support for the alternative model (Raftery, 1996; Burnham and Anderson, 2002, 2004). For the final model, we report parameter estimates and 95% credible interval. Other models, additional analyses, and further details of the robustness checks are reported in the ESM.

## Results

### Descriptive Statistics

**Figure 1:**
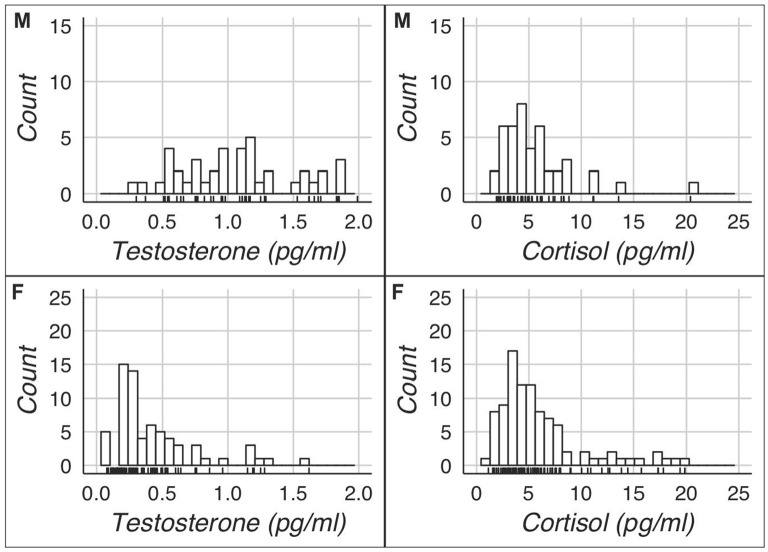
Hormonal distributions of men (M) and women (F).

**Figure 2:**
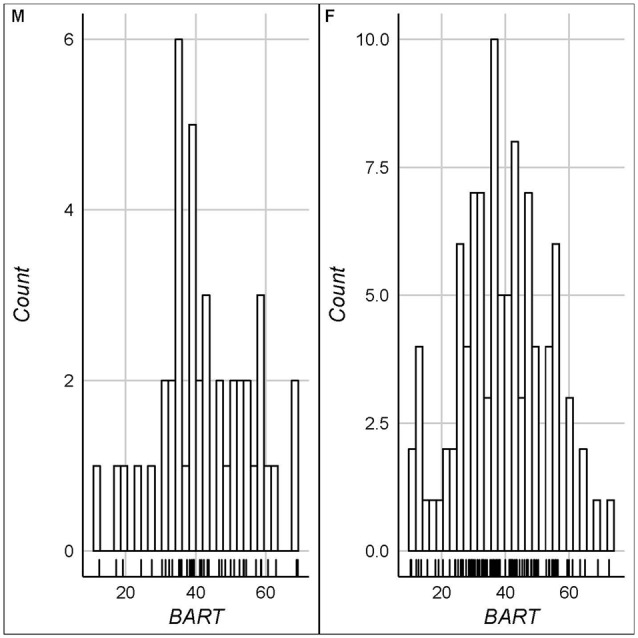
Histograms for balloon analog risk task (BART) scores (left panel: males/right panel: females).

The key descriptive statistics and baseline correlations can be found in Tables 1, 2. Figure 1 shows histograms for raw testosterone and cortisol levels. The medians were different between men and women for T (Mood’s median test: *p* < 0.0001), but not for C (Mood’s median test: *p* = 1). There were no extreme cases in hair testosterone concentrations for men, based on Tukey’s interquartile’s range (IQR) criterion (Tukey, 1977; Pollet and van der Meij, 2017). Whereas for women there were three extreme cases (>3 * IQR) in hair testosterone concentrations. For hair cortisol concentrations, there was one extreme value in the male data and three extreme values in the female data. Where relevant we reported the results with and without these extreme cases. Figure 2 shows the distribution of the BART scores.

**Table 1:** Means, standard deviations and correlations with confidence intervals for male sample.

Variable	*M*	*SD*	1	2	3	4	5
1. Testosterone	1.10	0.47					
2. Cortisol	5.64	3.49	0.29			
			[−0.01, 0.54]			
3. Left hand 2D:4D	0.96	0.03	−0.25	0.01			
			[−0.51, 0.05]	[−0.29, 0.31]			
4. Right hand 2D:4D	0.95	0.03	−0.28	−0.37*	0.49**	
			[−0.54, 0.02]	[−0.60, −0.08]	[0.23, 0.69]	
5. Overconfidence	12.10	12.52	−0.08	−0.06	0.09	−0.20	
			[−0.37, 0.23]	[−0.35, 0.25]	[−0.22, 0.38]	[−0.47, 0.10]	
6. Risk taking	43.48	12.46	−0.28	−0.12	0.08	0.21	−0.12
			[−0.54, 0.02]	[−0.41, 0.19]	[−0.23, 0.38]	[−0.10, 0.48]	[−0.41, 0.19]

Note. M and SD are used to represent mean and standard deviation, respectively. Values in square brackets indicate the 95% confidence interval for each correlation. *p < 0.05, **p < 0.01.

**Table 2:** Means, standard deviations and correlations with confidence intervals for female sample.

Variable	*M*	*SD*	1	2	3	4	5	6
1. Testosterone	0.35	0.31					
2. Cortisol	6.28	4.86	0.07					
			[−0.13, 0.26]					
3. Left hand 2D:4D	0.98	0.04	−0.05	0.03			
			[−0.25, 0.15]	[−0.17, 0.23]			
4. Right hand 2D:4D	0.97	0.03	−0.11	−0.06	0.76**			
			[−0.30, 0.09]	[−0.26, 0.14]	[0.66, 0.83]			
5. Overconfidence	11.69	11.67	0.07	−0.15	−0.04	0.06	
			[−0.13, 0.27]	[−0.34, 0.05]	[−0.24, 0.16]	[−0.14, 0.26]	
6. Risk taking	38.52	13.50	−0.03	−0.00	−0.02	−0.09	0.01	
			[−0.23, 0.17]	[−0.20, 0.20]	[−0.22, 0.18]	[−0.28, 0.12]	[−0.19, 0.21]	
7. Hormonal contraception use	0.70	0.46	−0.22*	−0.09	0.03	0.11	−0.09	−0.23*
			[−0.40, −0.02]	[−0.28, 0.12]	[−0.18, 0.22]	[−0.10, 0.30]	[−0.28, 0.11]	[−0.41, −0.03]

Note. M and SD are used to represent mean and standard deviation, respectively. Values in square brackets indicate the 95% confidence interval for each correlation. *p < 0.05, **p < 0.01.

### 2D:4D Ratio and Testosterone

None of the models provided substantial support for an effect of 2D:4D ratio on hair testosterone concentrations (all models ΔWAIC and ΔLOOIC < 2.1). In both males (*r*_left hand_ = −0.25; *r*_right hand_ = −0.28) and females (*r*_left hand_ = −0.05; *r*_right hand_ = −0.11), our data thus offer no support for a digit ratio effect on baseline testosterone. We acknowledge that the size of our male sample limits the robustness of this test and we cannot rule out the possibility of a small to moderate effect being undetected in our analysis. The correlations for both females and males are directionally consistent with such expectations.

### Bart Scores

In women, none of the models substantially supported an effect beyond the null model. The only exception was a model containing an effect of oral contraceptive use (ΔWAIC: 3.52 and ΔLOOIC: 3.52). This model suggests that those who take hormonal contraceptives have lower BART scores (*B* = −6.65 ± 2.85; 95%CI: −12.24 to −1.00).

**Figure 3:**
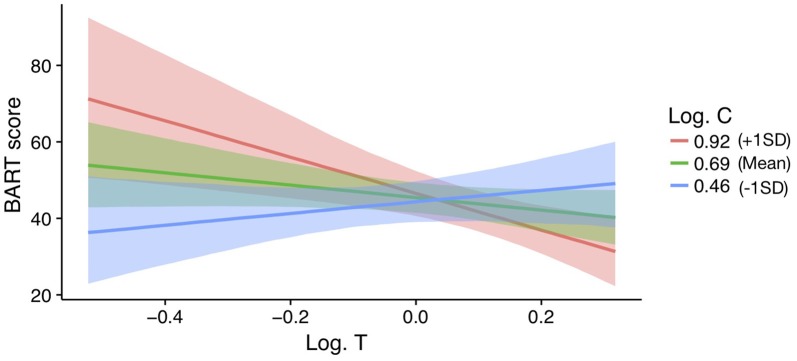
Effect of log transformed testosterone (Log T) on BART scores for varying levels of Alog transformed cortisol (Log C) in men (*z*-scored). Bands represent 95% confidence intervals.

In men, a model with a testosterone by cortisol interaction on BART scores is supported above the null (ΔWAIC = 3.73 and ΔLOOIC = 3.45). No other models were supported beyond the null. The parameter estimates, SE, and 95%CI for the testosterone by cortisol interaction model are reported in Table 3 (see ESM for further details on the model). The interaction effect is plotted in Figure 3. For those men low in cortisol, testosterone had a positive effect on their BART scores. In contrast, for those men high in cortisol, testosterone was negatively related to BART scores (*β*_interaction_ = −0.44 ± 0.16; 95%CI: −0.76 to −0.11). For women, there is no evidence for such an interaction effect (women: *B* = 21.04, 95%CI: −13.6 to 55.19) and, if anything, it runs in the opposite direction of the male effect (men: *B* = −135.84, 95%CI: −234.07 to −35.61).

**Table 3:** Parameter estimates, standard errors and 95%CI for T*C interaction model.

	Estimate	SE	95% lower	95% upper
Intercept	42.01	6.22	29.74	54.09
Log T	77.82	34.09	10.29	145.24
Log C	4.95	8.73	−11.96	21.93
Log T*Log C	−135.84	49.5	−234.07	−35.61

*Note. Log T = log transformed testosterone and log C = log transformed cortisol*.

We performed numerous, pre-specified robustness checks to further examine the results for men. Exclusion of the extreme case for cortisol did not alter our conclusions (*B*_interaction_ = −148.85 ± 50.97; 95%CI: −246.51 to −48.12). Similarly, controlling for age, BMI, or sexual orientation lead to the same conclusions (respective 95%CI for the interaction effect: −230.79 to −36.60; −251.18 to −31.66; −231.18 to −46.95). Excluding four cases due to excessive alcohol consumption or hard drug use, also upheld the effect (95%CI: −260.97 to −55.76). Neither accounting for how often participants washed their hair in a week, nor the method of hair drying affected this conclusion (respectively 95%CI: −239.55 to −40.97 and −233.93 to −38.65). Finally, controlling for certain medication usage, use of allergy medication or a history of psychological disorder also did not alter the statistical conclusion (respectively: 95%CI: −232.86 to −37.62; −237.04 to −38.96; and −231.72 to −40.27). Thus, after a range of checks we find consistent support for a testosterone by cortisol interaction effect on BART in men.

### Overconfidence

None of the models provided substantial support for a relationship between overconfidence and hair testosterone (*r*_males_ = −0.08; *r*_females_ = 0.07) or cortisol concentrations (*r*_males_ = −0.06; *r*_females_ = −0.15), nor overconfidence and risk taking (*r*_males_ = −0.12; *r*_females_ = 0.01).

## Discussion

The present study reexamined the relationships between testosterone and risk taking, using an alternative assay procedure in which testosterone levels are assayed from hair samples. We did not find evidence for a relationship between hair testosterone concentrations, 2D:4D ratios, and risk taking. However, we did find evidence for the interacting effect of hair testosterone and cortisol concentrations on risk taking in men, albeit in a small sample. We acknowledge that our final sample size for males imposes limitations on our statistical power, thus tempering the strength of our conclusions^2^.

### Theoretical Implications

Our findings did not support a relationship between hair testosterone concentrations and risk taking. As our testosterone sampling aggregated across approximately 3 months of participants’ testosterone levels, this finding provides necessary (but insufficient) support for the predictions of the Challenge Hypothesis (Wingfield et al., 1990; Archer, 2006) and the Biosocial Model of Status (Mazur, 1985; Mazur and Booth, 1998), both of which specify dynamic bidirectional relationships between socially driven fluctuations in testosterone and behavior. Consistent with these theoretical perspectives, previous reports have focused on context driven relationships between testosterone and risk taking (Coates and Herbert, 2008; Ronay and von Hippel, 2010), and while other studies have not specifically identified context as a factor, they have nonetheless measured testosterone and risk taking at a single time point, and examined the relationship between them at that moment in time (Apicella et al., 2008; Sapienza et al., 2009; Stanton et al., 2011). Previous results have been inconsistent, with positive (Apicella et al., 2008) and null relationships (Zethraeus et al., 2009). While it is possible that the positive effects in these studies are due to false positives, and the null effects perhaps the result of a weak relationship that is not captured by small sample sizes, or inconsistencies in the operationalization of risk taking, we speculate that the evidence for a relationship between testosterone and risk taking appears to be bound to the *activating* effects of the hormone within a specific context.

However, qualifying this speculative conclusion, we did find evidence in support of the Dual (hair) Hormone Hypothesis (Mehta and Josephs, 2010), albeit only in men and with a relatively small sample size (*n* = 53). Mehta and Josephs (2010) first articulated the possibility that the moderating role of cortisol might be due to low cortisol facilitating social approach, thus allowing for the overt expression of dominant (and perhaps risky) behaviors. However, due to cortisol’s effects on stress and social inhibition, higher testosterone may decrease dominance (and perhaps risky) behavior when cortisol is high. Those interested in reviewing the existing evidence for the Dual Hormone Hypothesis might read Mehta and Prasad (2015). In the current study we found that for men, hair testosterone concentrations were positively related to risk taking, only when levels of hair cortisol concentrations were low. When hair cortisol concentrations were high, we observed a negative relationship between testosterone and risk taking. Thus, although it has been suggested that one possibility for the few null findings surrounding the Dual Hormone Hypothesis might be that such effects emerge in response to social contextual primes (Mehta and Prasad, 2015), our data suggest this is not the case. Specifically, our data help clarify the Dual Hormone Hypothesis by demonstrating that the relationship between risk taking and the combination of high testosterone and low cortisol is not isolated to a time specific social context. Rather, we find that hormone levels, synthesized across a period of 3 months prior to completing a behavioral measure of risk taking, interact to predict risk taking behavior in a theory consistent manner.

Contributing to the lack of evidence for a relationship between circulating testosterone and 2D:4D ratio, we find no evidence for a relationship between hair testosterone concentrations and 2D:4D ratio. While further research is warranted before strong conclusions are drawn, we suggest this is an important null effect within the context of the ongoing discussion in the literature regarding the relationship between second to fourth digit ratio and circulating testosterone (Hönekopp et al., 2007). Aggregating testosterone levels across 3 months via hair samples filters out contextual noise in hormone measurements, so providing a stronger test of the relationship between testosterone and 2D:4D ratio. Taken together, the evidence suggests that both state-based levels of testosterone—such as are derived from single time point measures—and more stable aggregated levels of baseline testosterone—such as we captured via hair sampling—appear to be unrelated to second to fourth digit ratios. Future research might however explore the possibility of an interaction between 2D:4D ratio and hair testosterone concentrations, as previous research has reported that the effects of testosterone administration on women’s cognitive empathy are moderated by 2D:4D ratio (Van Honk et al., 2011).

Furthermore, despite theoretical suggestions of a relationship between testosterone and overconfidence (Johnson et al., 2006), we find no empirical support for this relationship with hair testosterone concentrations. This null effect is consistent with previous research (Ronay et al., 2017) that assayed testosterone concentrations from saliva samples.

Finally, we also found that hair cortisol concentrations were unrelated to overconfidence and risk taking. This finding is in line with other research showing that hair cortisol concentrations were unrelated to risk taking in behavioral tasks (Chumbley et al., 2014; Ceccato et al., 2016). However, only in men, Ceccato et al. (2016) did find a trend between higher hair cortisol concentrations and more investment in a gambling task. Furthermore, our null findings are not in line with research showing that high levels of conscientious, which are related to less risk taking behavior (Strickhouser et al., 2017), were related to smaller hair cortisol concentrations (Steptoe et al., 2017).

### Limitations and Future Directions

We acknowledge several limitations that serve as avenues for future research. First, although the total sample size is relatively large compared to other hair sample studies (e.g., Iglesias et al., 2015; Dettenborn et al., 2016), the number of men in our sample was relatively small. As the behavioral effects of testosterone are known to differ between men and women (e.g., Turanovic et al., 2017), future studies should replicate our findings in a more balanced gender sample. Second, Ribeiro et al. (2016) have shown that indirect finger length measures (from scans or photos) result in lower 2D:4D ratio scores than direct measures. Further work is needed in order to clarify whether the effect sizes of 2D:4D ratios are dependent on measurement protocol. Third, although the BART measure is an often used measure of risk taking (Lejuez et al., 2002), the measure could be confounded with participants’ beliefs about the choices and outcomes of others in the experiment (because of the cash prize). Although no computer task can perfectly simulate naturally occurring risk taking behaviors, the BART does simulate risk situations in a natural environment and has been shown to predict a number of real-world risk taking behaviors (Lejuez et al., 2002, 2003; Hopko et al., 2006). Furthermore, it allows for the assessment of an overall propensity for risk taking rather than the likelihood of engaging in a particular type of risk taking behavior, as is often case with self-report measures of risk-related constructs. Nevertheless, future studies should test the generalizability of the results to real-world situations. Fourth, our evidence suggests that both state-based levels of testosterone and baseline testosterone appear to be unrelated to 2D:4D ratios. This does not, however, rule against the possibility that 2D:4D is indeed a putative marker of prenatal testosterone exposure, and so lends itself to exploring the organizing effects of testosterone on behavior (Hönekopp et al., 2007).

## Author Contributions

RR and LM designed the study and collected the data. TVP conducted the analyses. All authors (RR, LM, JKO and TVP) contributed to the writing of the manuscript.

## Conflict of Interest Statement

The authors declare that the research was conducted in the absence of any commercial or financial relationships that could be construed as a potential conflict of interest.

## Footnotes

^1^The use of difference scores has received widespread criticism as difference scores are unreliable and tend to be confounded with variables that constitute the index (e.g., Cronbach and Furby, 1970; Cohen et al., 2003). Scholars have suggested regressing participants’ actual performance onto their self-evaluations and retaining the residuals of the self-evaluations (e.g., John and Robins, 1994).

^2^One reviewer requested a “traditional” frequentist power analysis (see ESM–Supplementary analysis: frequentist power analysis). This analysis showed that based on our sample size and with a power of 0.80 and a *p* level of 0.05, we were able to detect estimates of *f*^2^ = 0.099 and 0.254 for the female and male sample respectively. Cohen (1988) suggests interpretations of 0.02, 0.15 and 0.35 as small, moderate and large. *f*^2^ is a standardized measure of effect size.
